# Supplementary material for: Synthesis of Ultrastable Gold Nanoparticles as a New Drug Delivery System
Source: Molecules. 2019 Aug 13;24(16):2929. doi: 10.3390/molecules24162929 (PMC6720288; doi:10.3390/molecules24162929)
Supplement: Supplementary file 1 [file molecules-24-02929-s001.pdf]

Synthesis of ultrastable gold nanoparticles as a new drug delivery system

*Florence Masse, Pascale Desjardins, Mathieu Ouellette, Camille Couture, Mahmoud Mohamed Omar, Vincent Pernet, Sylvain Guérin and Elodie Boisselier**

Supplementary Materials

Content: **Page**

Table S1. Wavelength and absorbance of plasmon bands of GNP 800, GNP 2000 and GNP 6000, GNP NUS and GNP CIT in all conditions **2**

Figure S1. Absorbance at 515 nm of different concentrations of GNP 800, GNP 2000 and GNP 6000 **3**

Figure S2. Absorbance at the wavelength corresponding to the plasmon band maximum of GNP 800, GNP 2000 and GNP 6000 solubilized in PBS over time **4**

Figure S3. UV-visible spectrum and TEM image of GNP 800 in full size **4**

Figure S4. Absorbance of the supernatant at 514 nm according to the number of magnetizations of the magnetic beads **5**

Figure S5. Calibration curve of bimatoprost by HPLC (R^2 = 0.99) **5**

Table S1. Wavelength and absorbance of plasmon band maxima of GNP 800, GNP 2000 and GNP 6000, GNP NUS and GNP CIT in all conditions (associated to UV-visible spectra of Figure 1).

		Before treatment	Freeze drying	Heating	Ultra-centrifuge	Autoclave	Salts
GNP 800	Wavelength (nm)	515	514	515	515	520	515
	Absorbance (a.u.)	0.45	0.45	0.47	0.45	0.44	0.46
GNP 2000	Wavelength (nm)	514	514	515	514	520	515
	Absorbance (a.u.)	0.31	0.29	0.30	0.31	0.32	0.32
GNP 6000	Wavelength (nm)	514	515	514	514	520	515
	Absorbance (a.u.)	0.34	0.34	0.34	0.34	0.34	0.35
GNP NUS	Wavelength (nm)	520	520	520	520	525	520
	Absorbance (a.u.)	0.54	0.58	0.56	0.55	0.48	0.43
GNP CIT	Wavelength (nm)	521	n/a	521	n/a	520	n/a
	Absorbance (a.u.)	0.09	n/a	0.09	n/a	0.07	n/a

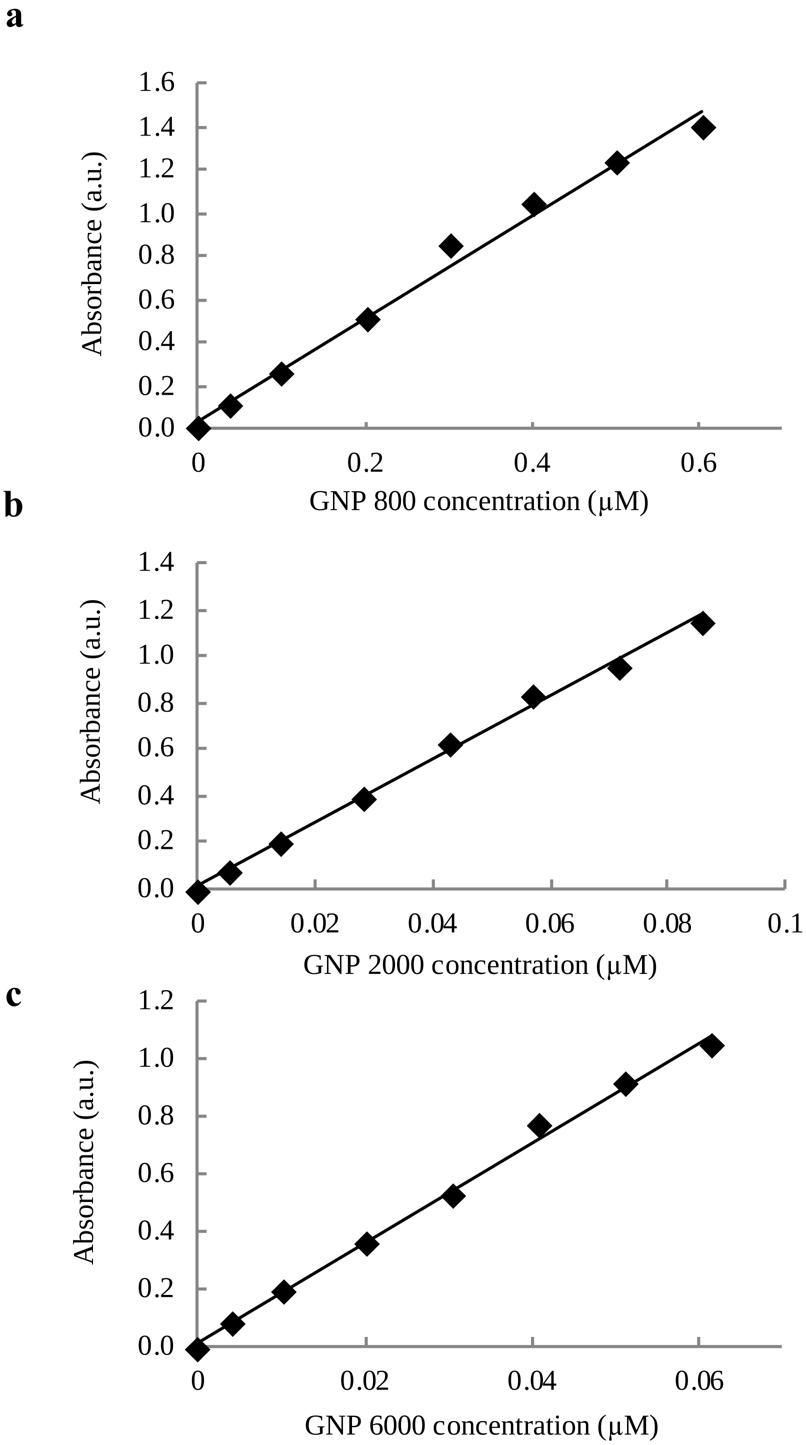

Figure S1. Absorbance at 515 nm of different concentrations of a) GNP 800, b) GNP 2000 and c) GNP 6000.

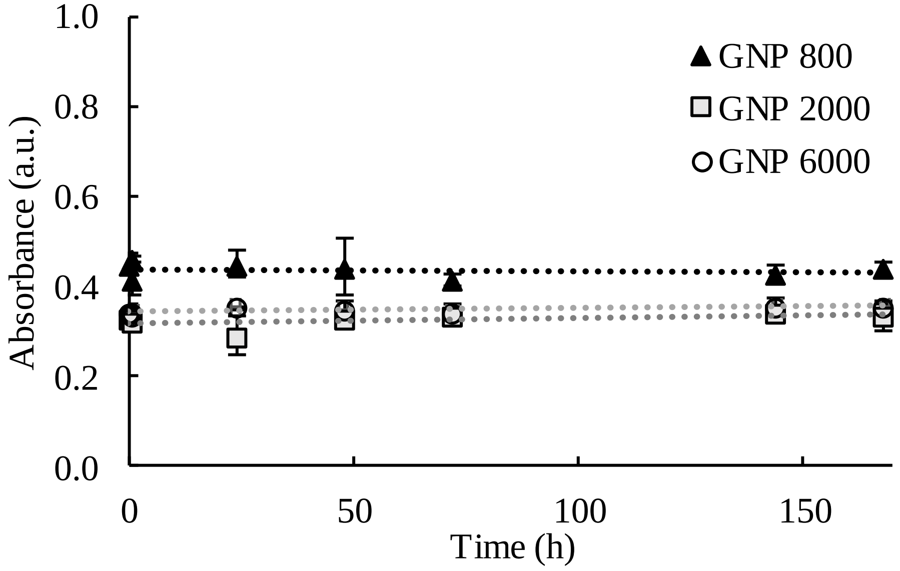

Figure S2. Absorbance at the wavelength corresponding to the plasmon band maximum of GNP 800, GNP 2000 and GNP 6000 solubilized in PBS over time.

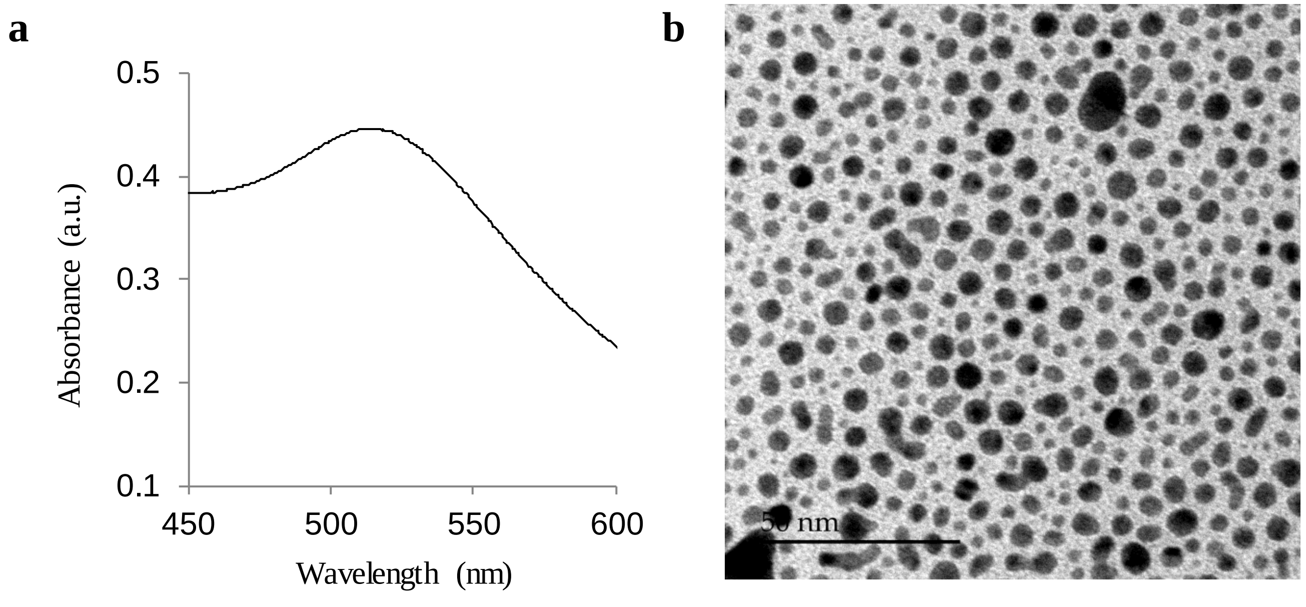

Figure S3. UV-visible spectrum and TEM image of GNP 800 in full size. The scale bar on the TEM image is 50 nm.

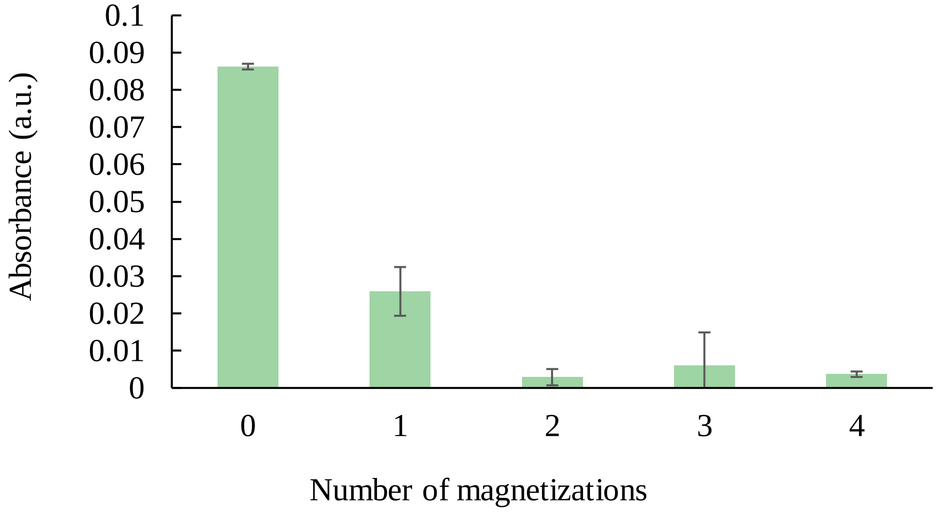

Figure S4. Absorbance of the supernatant at 514 nm according to the number of magnetizations of the magnetic beads.

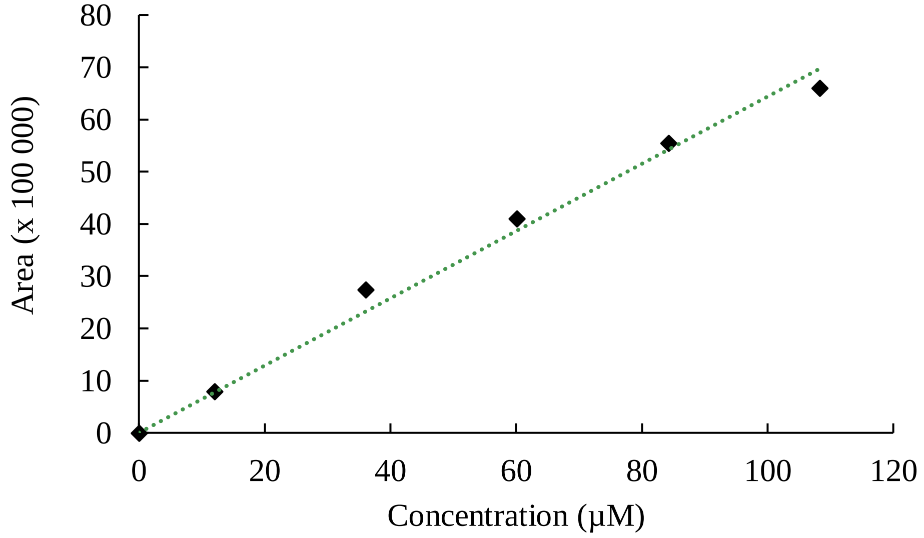

Figure S5. Calibration curve of bimatoprost by HPLC (R^2 = 0.99).
